# M. Roux

**Published:** 1854-07

**Authors:** 


### M. ROUX.

The great surgeon, Roux, died at Paris, on the 24th of March, at the age of 73 years. On the death of Dupuytren he became the senior surgeon of *Hôtel Dieu*, where he has been long distinguished above any surgeon of the day for the boldness of his operations. "M. Roux," his pupils were wont to say, "has performed upon the human body every operation but amputation of the *capital extremity.*" At his funeral six discourses were pronounced—one by Velpeau, in the name of the Institute; one by Malgaigne, for the Faculty of Medicine; one by F. Dubois, in the name of the Academy of Medicine; one by Marjolin, for the Society of Surgery; one by the venerable Larrey, for the Military Surgeons; and one by Duchaussoy, in the name of the Hospital Internes. We give an extract from the oration of Velpeau, being the apostrophe with which the eloquent Frenchman concludes his eulogy:

"Venerated master, faithful friend, thou hast nothing to fear from the celestial life, thou hast done thy duty toward thy fellow-creatures, thy career has been fully, nobly performed? Thy memory will never perish; thy name, ever honored, will occupy a great and illustrious page in the history of useful, of learned men. From the abode of the elect, God will permit thy great shade occasionally to cast an indulgent look on us; then thou wilt know that among thy former students, there is one who will cherish thy remembrance, and be grateful to thee to his last hour; who, in bidding thee an eternal adieu, leaves thy grave, his heart bursting with tears and sorrow."

The disease which terminated the life of M. Roux was apoplexy. He was attacked while shaving himself to go to a ball, on the evening of the 28th of January, but lingered until the 24th of March, when he expired.

